# Implication of metabolism in the polarization of tumor-associated-macrophages: the mass spectrometry-based point of view

**DOI:** 10.3389/fimmu.2023.1193235

**Published:** 2023-07-12

**Authors:** Giulia De Simone, Cristiana Soldani, Aurelia Morabito, Barbara Franceschini, Fabrizio Ferlan, Guido Costa, Roberta Pastorelli, Matteo Donadon, Laura Brunelli

**Affiliations:** ^1^ Laboratory of Metabolites and Proteins in Translational Research, Istituto di Ricerche Farmacologiche Mario Negri IRCCS, Milan, Italy; ^2^ Department of Biotechnologies and Biosciences, Università degli Studi Milano Bicocca, Milan, Italy; ^3^ Hepatobiliary Immunopathology Laboratory, IRCCS Humanitas Research Hospital, Milan, Italy; ^4^ Department of Electronics, Information and Bioengineering, Politecnico di Milano, Milan, Italy; ^5^ Department of Biomedical Sciences, Humanitas University, Milan, Italy; ^6^ Department of Hepatobiliary and General Surgery, IRCCS Humanitas Research Hospital, Milan, Italy; ^7^ Department of Health Sciences, Università del Piemonte Orientale, Novara, Italy; ^8^ Department of General Surgery, University Maggiore Hospital, Novara, Italy

**Keywords:** tumor associated macrophages (TAMs), prognostic markers, metabolism, mass spectrometry, TAM polarization

## Abstract

Tumor-associated macrophages (TAMs) represent one of the main tumor-infiltrating immune cell types and are generally categorized into either of two functionally contrasting subtypes, namely classical activated M1 macrophages and alternatively activated M2 macrophages. TAMs showed different activation states that can be represent by the two extremes of the complex profile of macrophages biology, the M1-like phenotype (pro-inflammatory activity) and the M2-like phenotype (anti-inflammatory activity). Based on the tumor type, and grades, TAMs can acquire different functions and properties; usually, the M1-like phenotype is typical of early tumor stages and is associated to an anti-tumor activity, while M2-like phenotype has a pro-inflammatory activity and is related to a poor patients’ prognosis. The classification of macrophages into M1/M2 groups based on well-defined stimuli does not model the infinitely more complex tissue milieu where macrophages (potentially of different origin) would be exposed to multiple signals in different sequential order. This review aims to summarize the recent mass spectrometry-based (MS-based) metabolomics findings about the modifications of metabolism in TAMs polarization in different tumors. The published data shows that MS-based metabolomics is a promising tool to help better understanding TAMs metabolic phenotypes, although it is still poorly applied for TAMs metabolism. The knowledge of key metabolic alterations in TAMs is an essential step for discovering TAMs polarization novel biomarkers and developing novel therapeutic approaches targeting TAM metabolism to repolarize TAMs towards their anti-tumor phenotype.

## Introduction

Macrophages are immune cells essential component of the innate immune system, with a wide distribution in lymphoid and non-lymphoid tissues throughout the body that play a pivotal role in innate immunity, tissue homeostasis, and response to adverse signals, such as pathogenic infections or inflammatory stimuli (1–3).

Macrophages principally originate from monocyte precursors mainly generated from hematopoietic stem cells placed in the bone marrow; then, monocytes migrate to several tissues and differentiate into tissue-specific macrophages (3, 4). Instead, tissue-resident macrophages such as Kupffer cells (liver), microglia (central nervous system), and Langerhans cells (skin) originate from the yolk sac and fetal liver during primitive and definitive hematopoiesis and they are responsible for the innate immunity (5, 6).

**Figure 1:**
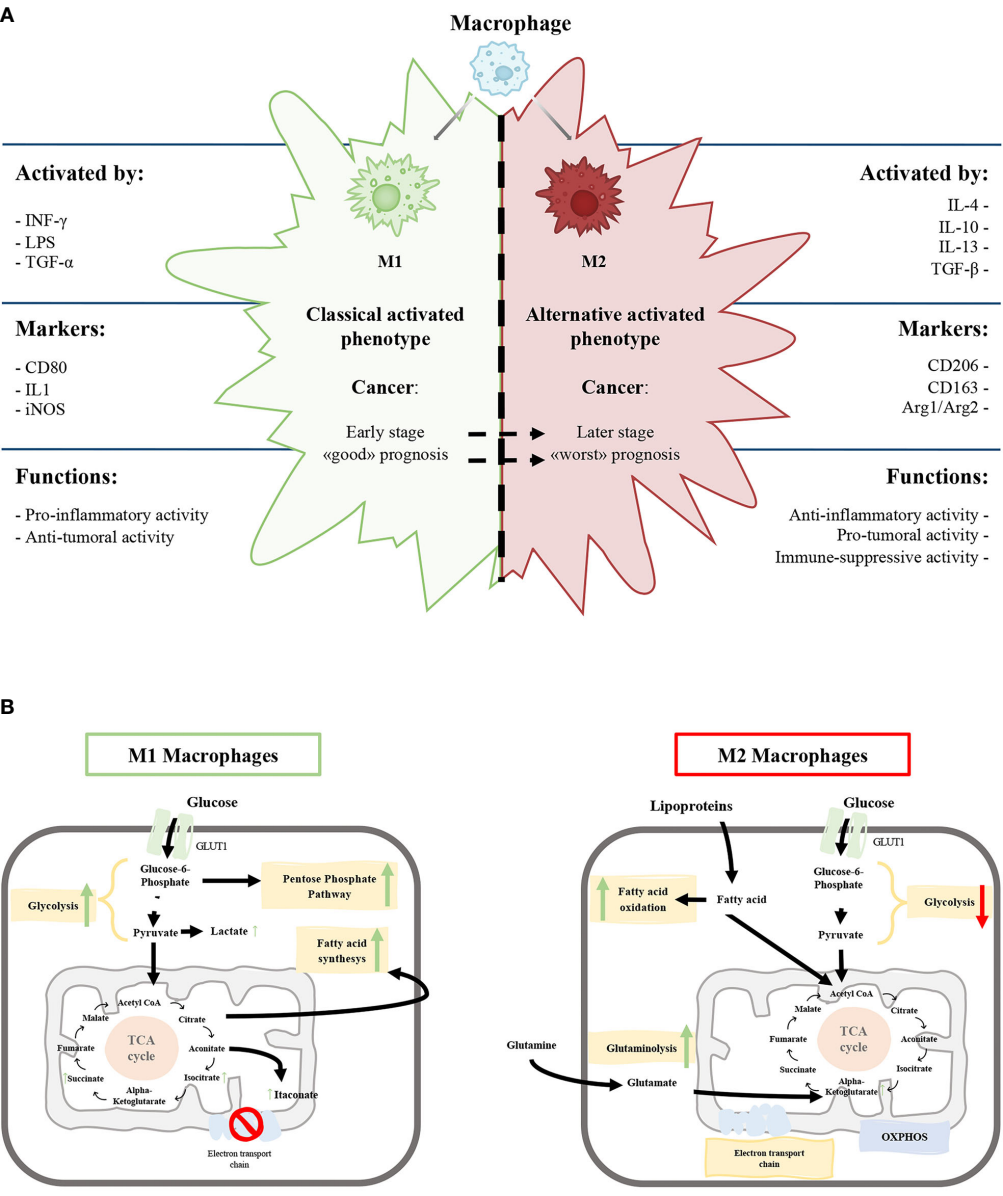
**(A)** Schematic representation of the characteristics of M1 and M2 macrophages phenotypes. **(B)** Schematic view of M1 and M2 macrophages metabolism. Green and red arrows indicate an up-regulation and down-regulation of a specific pathway respectively.

Circulating monocytes are recruited by chemotactic signals generated upon injury, infection etc. and migrate to the target tissues where they differentiate and polarize into mature macrophages depending on the microenvironment (5, 7, 8). It is possible to identify two main activation states that represent the two extremes of the complex profile of macrophage biology (9). The current classification into M1-type (classically activated macrophage) and M2-type (alternatively activated macrophage) is based upon macrophage polarization and describe macrophages different behaviors (10). One phenotype, the pro-inflammatory or classically activated M1 phenotype, allows the host to fight infections and pathogens and exhibits anti-tumoral activity. The second phenotype, the anti-inflammatory or alternatively activated M2 phenotype, displays the capability to repair damaged tissues but also presents a pro-tumoral functions (**Figure 1A**) (11–15).

The M1 phenotype is induced by microbial products like lipopolysaccharide and cytokines secreted by Th1 lymphocytes, such as Interferon-γ (IFN-γ) and tumor necrosis factor-alpha (TNF-α). On the other hand, anti-inflammatory molecules, such as glucocorticoid hormones and interleukin (IL)-4, IL-10, IL-13, secreted by Th2 lymphocytes, induce the M2 phenotype (**Figure 1A**) (5, 16, 17).

M1 macrophages express high levels of inducible nitric oxide synthetase (iNOS) that promote the release of nitric oxide, reactive oxygen species and pro-inflammatory cytokines (IL-1β, IL-6, IL-12) promoting Th1 response (2, 13). The high production of anti-microbial and anti-tumoral molecules gives to M1 macrophages the capability to kill intracellular pathogens (5, 18). Classically activated macrophages express specific surface proteins, such as CD68, CD80, and CD86 (**Figure 1A**).

In contrast, M2 macrophages produce a high amount of anti-inflammatory cytokines (IL-10, TGF-β), low levels of pro-inflammatory cytokines (IL-12) and diminished expression of the NOS enzyme. M2 macrophages have a strong phagocytosis capacity and induce angiogenesis and lymphangiogenesis (2, 19, 20). Furthermore, the arginase pathway induces increased production of ornithine, collagen, and polyamines with an elevated expression of arginase-1 (Arg1) (**Figure 1A**).

From a biochemical point of view, the metabolism of M1 and M2 activated macrophages were extensively characterized. M1 macrophages metabolism is based on anaerobic glycolysis, truncated tricarboxylic acid cycle (itaconate production), fatty acid synthesis and pentose phosphate pathway (**Figure 1B**) with, a consequent decrease in oxidative phosphorylation (OXPHOS) and fatty acid oxidation (5, 21–23)

M2 macrophages have a poorly glycolytic profile with higher arginine metabolism and preferential fatty acid oxidation (oxidative glucose metabolism), glutamine metabolism and OXPHOS (**Figure 1B**) (5, 18, 19, 24). Glycolysis is not essential for M2 phenotype, and glucose is mainly employed to sustain OXPHOS (25–27).

In tumors, monocytes from bone marrow and circulating monocytes are recruited through the release of cytokines and macrophages were functionally polarized in tumor-associated macrophages (TAMs) (9, 28). Within the tumor microenvironment (TME), TAMs exhibit high plasticity and undergo specific functional metabolic alterations according to the availability of tumor tissue oxygen and nutrients, thus further contributing to tumorigenesis and cancer progression.

In this review, we describe the relationship between TAMs metabolic profile and activation states and their involvement in cancer pathologies. We focus on mass spectrometry (MS)-based metabolomics analysis that is able to detect, quantify and map a huge number of molecules for recapitulate the different TAMs metabolic phenotypes (29).

## TAMs density and polarization as prognostic marker in cancer pathologies: state of the art

TAMs are one of the most abundant immune myeloid populations in the TME (30). Meta-Analyses indicates that high density of TAM (CD68+) could be associated with worse prognosis in almost all cancers (31–35), contradictory in gastric cancer (32, 36) or should be linked to a favorable prognosis in colorectal cancer (37, 38).

TAMs could exhibit pro-tumoral or anti-tumoral roles depending on the stimuli generated by the TME and can acquire different functions and properties depending on cancer type, grade, and during tumor progression (39). Several studies are committed to quantify and characterize the M1 and M2 TAM phenotype to better refine the prognostic significance of TAMs.

### M1-like TAMs as prognostic marker

TAMs can show a M1-like phenotype, in particular during the early stages of tumor development (2, 20, 39, 40), promoting antineoplastic and phagocytose activity. During the tumor progression, the TME produce a large amount of growth factors and anti-inflammatory mediators that induce a shift in TAMs phenotype.

In ovarian cancer, two prospective observational studies (140 Italian and 112 Chinese patients) showed that intratumoral M1/M2 TAM ratio decreased as the cancer stage increased (41) with a significant association between higher M1/M2 TAMs ratio and the longer overall survival and progression-free survival (42),

Similar findings were obtained in Non-Small Cell Lung Cancer (NSCLC) studies, where high infiltration of M1 like TAMs (41 UK and 80 Lithuanian subjects) were associated with a better survival outcomes (43, 44). Consistently with these studies, a high density of iNOS+ M1-like macrophages predicted the improved survival rate in a cohort (40 Finland patients) of HER2+ breast cancer subjects (45). Also considering TAMs morphologies, M1-like macrophages more frequently appear round and flattened, as opposed to M2-like cells that present an elongated morphology. A higher percentage of TAMs with small dimensions and regular shape were associated with a better prognosis in colorectal liver metastasis patients (101 Italian patients) (46).

Overall, these data point out that M1-like TAMs infiltrate may be associated with a favorable survival rate in several cancer pathologies.

### M2-like TAMs as prognostic marker

The alternative activated M2-like phenotype promote cancer cells growth through the secretion of pro-angiogenic factors, like vascular endothelial growth factors, and immunosuppressive factors, as IL-10 and TGF-β (47). This M2-like phenotype is considered as pro-tumoral increasing neovascularization, angiogenesis, infiltration and consequently metastatization; has therefore an immune-suppressive activity and antitumor adaptive immune response (14, 16, 26, 48).

High number of M2-like TAMs were observed associated with tumor growth and poor patient prognosis in breast (562 Finland, 144 Sweden patients), NSCLC (349 Korean patients) and melanoma (184 Finland, 94 Italian patients) cancers (49–52).

Overall, these studies highlight that M1 and M2 TAMs may be useful markers of patient prognosis (M1-like better and M2-like worst) regardless of tumor type and patient ethnicity. However, meta-analyses considering prospective studies employing standardized methodology and larger sample sizes are still needed to validate the prognostic significance of M1 and M2 TAMs infiltration.

Metabolism as modulator of macrophage functionality driving the balance between M1-like and M2-like TAMs might lead to different strategies for chemotherapeutic and/or immunotherapeutic approaches.

## Mass spectrometry-based metabolomics approaches in cancer research

Metabolomics is the large-scale study of endogenous small molecules of low molecular weight molecules (<1000 Da), commonly known as metabolites, within cells, biofluids, tissues, or organisms. Metabolomics together with genomics, transcriptomics, and proteomics, drive the connection between the genotype and the phenotype in both physiological and pathological processes in order to be a powerful tool in biomarker discovery, developing drugs, and personalized medicine (53–55). Metabolomics is an essential tool for the simultaneous measurement and quantification of thousands of small metabolites from biological matrices, with the possibility to be hypothesis-generating, without *a priori* knowledge, or hypothesis-driven, with *a priori* knowledge about the metabolites present in the sample. The applicability of metabolomics in identifying metabolic dysregulation has been demonstrated in a wide range of human diseases, including cardiovascular diseases, diabetes, obesity, and cognitive disorders, but also cancers (54). For this reason, metabolomics could be considered a promising approach for the identification of metabolites acting as biomarkers to allow early diagnosis, to follow pathological processes and progression or response to treatments.

The two main analytical techniques employed for metabolomics profiling are Nuclear Magnetic Resonance (NMR) and Mass Spectrometry (MS), which allow the achievement of both qualitative and quantitative information. We will focus on the role of MS as a powerful tool for the identification of a large set of ionized analytes based on their mass-to-charge ratio (*m/z*) (53, 56).

### Analytical methods for MS-based metabolomics

MS is usually coupled with a chromatographic separation system (hyphenated techniques), such as gas chromatography (GC)-MS, liquid chromatography (LC)-MS [both high-performance LC (HPLC) and ultra-performance LC (UPLC)], and capillary electrophoresis (CE)-MS, but a sample could be also directly injected into the MS (flow-injection analysis-MS) (56, 57). GC-MS is employed for the identification of volatile compounds, like fatty acids and organic acids, requiring a derivatization step before the analysis (58). Electron impact (EI) and chemical ionization (CI) sources are commonly used with a GC separation system (59). However, LC-MS does not require any derivatization and allows the detection of more analytes (60), and it is usually combined with an electrospray (ESI) and CI sources.

CE-MS allows the separation of polar and charged metabolites based on their electrophoretic mobility, an intrinsic property that depends on the size and charge of the molecule (61). To date, MS-based metabolomics strategies are widely employed in the tumor research area to identify biomarkers for prediction, diagnosis, and prognosis (62, 63).

### Metabolomics approaches

Untargeted and targeted MS are the two methods used for the identification of endogenous small molecules (58). The untargeted strategy allows the identification of a huge number of metabolites with high accuracy using high-resolution MS, without knowing basic information about the metabolome of the sample.

Orbitrap and Time-of-flight (ToF) are the preferred mass analyzers for high-resolution untargeted metabolomics analysis. Ionized molecules in the orbitrap analyzer are forced to move in complex spiral patterns by the electrostatic field, combining axial oscillations with rotation around the central axis (56). Different frequencies of oscillation are then associated with different *m/z*. In the ToF analyzer, ions with initial kinetic energy enter a field-free drift region of known length and are dispersed in time based on their different *m/z*. Ions with the larger *m/z* arrive at the end of the drift length after ions with a smaller *m/z* (64).

Then, after the final ion detection, the use of database is required for metabolites identification and annotation [HMDB (65), METLIN (66)]. This annotation is performed by comparing the experimental mass to libraries within a mass tolerance frame, depending on the mass spectrometer used for the analysis (58, 60, 67). On the other hand, the information should be incomplete cause of employed solvents, pH, chromatographic separation, ionization techniques; and the detection of chemical unknowns with no annotation in database. MS/MS fragmentation experiments are then required to confirm the metabolites’ structure, especially to distinguish co-eluting isobaric species (67).

Targeted metabolomics is a hypothesis-driven strategy that allows the identification of a specific set of metabolites from a panel of interest with high accuracy, selectivity and sensitivity (59).

Triple quadrupole (QQQ) and quadrupole ion trap are useful mass analyzers for targeted metabolomics (56, 58). QQQ is the most common type of tandem MS, the first and the third quadrupole are used as mass filters, while the second quadrupole is used as a collision cell to generate fragment ions. Into the Q, ions with a particular range of *m/z* values have stable periodic trajectories imposed by the direct current and the radio frequency potential of the four rod electrodes composing the Q that periodically changes in time (56, 68). The voltage polarity between two adjacent rods is opposite (64). The quadrupole is often combined with other mass analyzers to filter ions to remove matrix ions such as in Q-Trap.

The ion trap analyzer is a modification of the Q with improved sensibility. The operating principle of Q and ion trap is the same: ions of different *m/z* are selectively ejected from the analyzer by varying the radio frequency potential (68).

Targeted metabolomics allows the quantification and semi-quantification using internal standards (69) and the Multiple Reaction Monitoring (MRM) approach, performed on QQQ or Q-ion trap, is used for the exact identification of the molecules of interest, selecting specific precursor and product ions.

The number of publications in PubMed related to cancer metabolomics shows that the MS-based approach has been largely preferred over NMR with more than 4000 results obtained for the search query “*cancer metabolomics AND mass spectrometry*” and less than 1500 results obtained for the search “*cancer metabolomics AND NMR*”, supporting the higher impact of MS-based techniques on cancer research.

### MS-based metabolomics limitations and future perspective

Mass spectrometry (MS) techniques, because of their sensitivity and selectivity, have become methods of choice to characterize the human metabolome, being able to detect and quantify many thousands of metabolite features simultaneously. Because the metabolite composition is central to every living organism the in-depth biological insides, metabolomics can advance research across a variety of scientific areas. However many challenges still exist for a successful metabolomics study. Much has been discussed on this issue elsewhere (67, 70, 71), here we address few of the unique challenges for metabolomics. The first one is analytical, due to the chemical diversity of the metabolites, their wide dynamic range and the confidence for their unambiguous identification. To this purpose, implementation of analytical and bioinformatic tools for the accurate and standardized metabolites identification is a current goal to be achieve for a large-scale coverage of metabolites and to facilitate data processing. Although there is no single analytical platform strategy that provides the complete coverage for the whole metabolome, there are common experimental criteria to all strategies that need to be addressed. Robust metabolomics results hinge on further key elements such as a proper study design (e.g. number of observations, power size, and sampling storage), method optimization, data processing and final validation. The application of this virtuous workflow will help not only in assigning biological meaning to metabolites but also to move towards finding robust mechanism of diseases. Metabolomics as for the other omics, is not a separate discipline, and the implementation of bioinformatic algorithms and computational strategies for multi-omics integration is fundamental to place metabolites in a biological context and to associate metabolites with phenotype causality. This promotes the understanding of metabolome in a system-wide level in order to develop personalized treatments and help in early diagnosis of disease. It is believed that the continuous progress in technologies together with bioinformatics and computational tools will feed metabolomics research to guide not only biomarkers discovery but also to delve and discover mechanisms of disease development and progression.

## The MS-based metabolomics profile of TAMs in cancer pathologies

Exploring the biochemical mechanisms underlying TAMs polarization during tumor development and progression could contribute to the development of new therapeutic approaches. In the last few years, metabolism of M1 and M2-like macrophages was mainly studied in *in-vitro* polarized TAMs (72, 73). Nevertheless, the *in-vivo* metabolic phenotypes of resident TAMs directly isolated from fresh tumor specimens are not largely explored. Considering the heterogeneity of activation stimuli from TME, deciphering the TAMs metabolism in the tissue has a vital importance in cancer research.

We summarized the scientific analysis of TAMs metabolism obtained by MS-based metabolomics studies in different cancer pathologies. MS-based studies on TAMs metabolism could determinate the metabolic alterations in different metabolic pathways such as glycolysis, mitochondrial machinery, amino acids, and lipid metabolism. These information are critical for identifying new biochemical pathways underlying tumor resident TAMs polarization useful to influence overall patient survival.

### Central cellular biochemical pathways in TAMs

Untargeted metabolomics by using CE-ToF-MS of *in-vivo* sorted murine TAMs from a colon adenocarcinoma xenograft model in both early (14 days after tumor implantation) and late tumor stage (28 days after tumor implantation) showed an increasing of different cellular metabolic pathways (glycolysis, methionine metabolism, TCA cycle, and glutamine and glutamate metabolism) in the tumor resident TAMs relative to myeloid-derived suppressor cells (74). This metabolic asset [increase in oxidative phosphorylation (OXPHOS) and glycolysis] of TAMs was confirmed in peritoneal Mϕ macrophages isolated from naive and ID8 tumor-bearing mice (75). The untargeted metabolomics (GC-TOF) identified itaconic acid, the product of the catabolism of mitochondrial cis-aconitate, the most highly upregulated metabolites in Mϕ macrophages of tumor-bearing mice (75). The high glycolytic activity of TAMs was also observed by untargeted metabolomics (by means of UHPLC-Orbitrap) on *in-vitro* generated human pancreatic ductal adenocarcinoma (PDAC) TAMs. The mixed M1/M2 TAMs generated from monocytes exposed to PDAC conditioned medium were characterized by elevated glycolysis, increased lactate production and reduced OXPHOS compared to normal macrophages (76).

The reliance on glycolysis of pancreatic cancer derived TAMs was confirmed by untargeted metabolomics (using both LC-ToF-MS and CE-ToF-MS) on TAMs deleted for the Glucose Transporter 1 (GLUT1). Tumor resident GLUT1 deleted TAMs showed the decrement of glycolytic intermediates such as glucose-6-phosphate and fructose 1,6-biphosphate with no substantial difference of the TCA cycle relative to control (77).

For the characterization of metabolic pattern characteristic of M1 and M2-like TAMs, a metabolic flux analysis (13C-labeling experiments coupled with GC-MS) on *in-vivo* sorted NSCLC resident TAMs (3LL-R Lewis lung murine model) was used. In this model M1-like TAMs (major histocompatibility complex (MHC)-IIhi) display a hampered tricarboxylic acid (TCA) cycle, while M2-like TAMs (MHC-IIlo) show higher OXPHOS and glycolytic metabolism (78). Another metabolic profiling (through UPLC QQQ tandem MS) of *in-vitro* conditioned PDAC TAMs indicated that nucleoside metabolism is the principal biochemical pathway able to distinguish TAM and M2 macrophages from the M1 (72).

Metabolism of M1 was also studied in *ex vivo* NSCLC tumor slices. Slices were treated with the macrophage activator β-glucan, and metabolic changes were monitored by metabolic flux analysis (13C_6_-glucose LC–MS). MS highlighted a higher synthesis of itaconate and higher levels of NADPH in M1-like generated TAMs relative to the general TAMs population (79).

### Lipid metabolism in TAMs

Lipidomics is focused on the identification of lipid species present within a cell, organ, or biological system. The lipidome compartment in the cells is composed of several lipid categories (e.g., fatty acids, glycerolipids, sphingolipids, and sterol lipids). Among the metabolic alterations observed in TAMs, lipid metabolism seems to directly affected TAMs polarization and function (80).

Eicosanoids quantification (Target lipodomics by using LC-QQQ tandem MS) on *in-vivo* sorted tumor resident TAMs populations (resident alveolar macrophages MacA and M2-like TAMs MacB) in orthotropic lung cancer model, showed that M2-like TAMs had low level of eicosanoids production relative to resident alveolar macrophages (81).

Global lipidomics analysis (by using LC-HRMS with Orbitrap) on *in-vitro* generated TAMs (monocytes exposure to gastric cancer conditioned medium) showed almost 10-fold higher triacylglycerol levels relative to control macrophages (73). This data was also supported by the high amount of total lipid found in *in-vivo* TAM sorted from both murine and human gastric cancer specimens. The lipid accumulation seems to occur by uptakes of lipids released by cancer cells, that drives TAMs polarization toward an M2‐like profile (73). Finally, target lipidomics (by QTrap analyzer) on conditioned medium from *in-vivo* sorted TAMs reveal that M2-like TAMs (CD163+CD206+) actively released of PLA2G7 and autotaxin that play an essential role in ovarian cancer invasiveness and metastatic spread (82).

Overall, mass spectrometry-based metabolic profiling of TAMs converges to define the increased glycolytic and OXPHOS activity of TAMs relative to control macrophages also considering the *in-vivo* sorted and *in vitro* conditioned TAMs (**Table 1**). Considering the heterogeneity of the TAMs populations in the cancer microenvironment and the different experimental settings for determining TAMs subpopulation, it is difficult to speculate the attribution of the augmented metabolism at a particular TAMs populations. It is possible to speculate that M2-like TAMs identified as CD11b+Ly6G−Ly6ClowMHC-IIhigh (78) rely on glycolysis, mitochondrial machinery, and lipid metabolism to sustain their functionality (**Table 1**). On the contrary, M1-like TAMs, when compared to the general control macrophages cells, seem be less metabolic active with a hampered OXPHOS capacity (**Table 1**). These data only partially agree with the metabolic characteristics of M1, and M2 polarized macrophages. Indeed, the M2 phenotype is mainly defined by a poor glycolytic profile with higher arginine metabolism and preferential fatty acid oxidation (oxidative glucose metabolism) and OXPHOS (5, 19) (**Figure 1**).

**Table 1:** List of papers employing mass spectrometry based metabolomics approaches to describe the metabolic state of TAMs populations.

Study	Specimens	TAMs populations	Metabolic pathways	Trend of metabolic alterations
(74)	Subcutaneous colon adenocarcinoma mouse model	General macrophages (CD11b+ sorted cells)	Glycolysis TCA cycle Glutamine Methionine metabolism	Increased in CD11b+ resident tumor cells relative to the spleen resident cells)
(75)	Orthotropic mouse model of ovarian cancer	General macrophages (F4/80+ sorted cells).	Glycolysis Polyamines TCA cycle	Increased in resident peritoneal macrophages Mϕ of bearing tumor mice relative to peritoneal resident naive Mϕ
(76)	Human pancreatic ductal adenocarcinoma (PDAC) cell lines	Mixed M1, M2 phenotype (*in-vitro* conditioned monocytes CD68+, CD163+, M-CSFR, and CD206+)	Glycolysis	Increased in conditioned TAMs relative to control monocytes
(77)	Orthotropic mouse model of PDAC	General macrophages (CD45+ sorted cells)	Glycolysis	Increased in resident tumor CD45+ TAMs relative to the non-tumor bearing controls cells
(78)	Subcutaneous Lewis Lung carcinoma mouse model	M1-like and M2-like TAMs (CD11b+Ly6G−Ly6ClowMHC-IIlow and CD11b+Ly6G−Ly6ClowMHC-IIhigh sorted cells)	TCA cycle Glutamine metabolism	Increased in tumor resident MHC-IIhi TAMs relative to the Ly6ClowMHC-IIlow
(72)	Human PDAC cell lines	M2 macrophages (*in-vitro* conditioned murine bone-marrow-monocytes)	Pyrimidines	Increased in conditioned murine M2 like TAMs relative to control macrophages
(79)	Non-small cell lung cancer tumor slices	M1-like TAMs (iNOS and CD68+ stained cells)	Glycolysis TCA cycle	Increased in iNOS and CD68+ TAMs
(81)	Orthotropic Lewis lung mouse model	Mixed macrophages populations (SigF+/CD11c+/F480+/CD11b (MacA), F480+/CD11b+/Ly6G-/SigF-, (MacB) sorted cells)	Eicosanoids	Increased in MacA relative to MacB populations
(73)	Gastric cancer cell line	M2-like TAMs (*in-vitro* conditioned CD206, CD163, TGFβ, Arg‐1 murine bone-marrow cells)	Triacylglycerol	Increased in conditioned TAMs relative to control
(82)	High-grade serous adenocarcinoma	General macrophages (EpCAM+ CD14+ CD163+ CD206+ sorted cells)	Lysophosphatidic acid	Increased in TAMs populations

For each paper, the reference, the specimens, the TAMs populations selected in the study, the altered metabolic pathway and the trend of deregulation.

## Discussion

TAMs are one of the most common immune cells in the TME and they are characterized by a great plasticity and adaptability to the environment they infiltrate by re-programming their activation state towards a pro- (M1) or an anti- (M2) inflammatory phenotype (26). Macrophages polarization in various malignancies appears to be complex, and the M1 to M2 phenotype switching can occur during cancer progression. M1-like and M2-like TAMs can be co-expressed inside the tumor environment and their ratio can change during cancer progression. A high amount of M1-like TAMs is typical of the early stages of tumors development and it is associated with good prognosis in several malignancies, while M2-like TAMs have a strong correlation with worst prognosis and advanced tumor stage. Understanding and deciphering the complexity of metabolic mechanisms involved in TAMs polarization could be a useful strategy to target cancer. Mass spectrometry-based metabolomics/lipidomics provide the capability to profile and detail the metabolic alterations in TAMs providing new mechanistic hypothesis for find more effective TAMs-targeted therapy. The collected results point to the fact that there are few articles dissecting the metabolic characteristics of the *in-vivo* derived human TAMs populations and up to now it is only possible to state that *in-vivo* patient derived TAMs populations (mixed M1- M2-like phenotypes) had an augmented metabolic activity that not completely recapitulate the M1, M2 macrophages metabolic peculiarity. Of note, current evidence based on MS-metabolomics studies are still limited and mostly focused in comparing TAMs metabolism relative to non-tumor counterparts, thus not allowing an in-depth metabolic characterization of TAMs subset. So far, metabolic profiling is mainly related to the general description of metabolic pathways and does not go deeply into the definition of the enzyme activity to discover new fragile metabolic points that might be pharmacologically targeted. Considering the technological advances in the field of cytofluorimetry and mass spectrometry, it is urgent and feasible to investigate the metabolic structure of TAMs sorted directly from patients, without having to analyze orthotropic mouse models or *in vitro* conditioned monocytes.

Therefore, more effort is needed to implement bulk metabolic analyses such as targeted metabolomics, 13C-label-tracing, and extracellular flux analysis to provide the complete metabolic picture and address the therapeutic implications of TAMs metabolism.

## Author contributions

Conceptualization, all authors. Project administration, MD. Resources, MD. Supervision, LB. Writing original draft, GD and LB. Review and editing, all authors. All authors contributed to the article and approved the submitted version.
